# Myogenic temporomandibular disorders: Clinical systemic comorbidities in a female population sample

**DOI:** 10.4317/medoral.21249

**Published:** 2016-10-01

**Authors:** Miguel de-Pedro-Herráez, Juan Mesa-Jiménez, César Fernández-de-las-Peñas, José-Luis de-la-Hoz-Aizpurua

**Affiliations:** 1 DMD, MS. Master’s Degree Program in Orofacial Pain and Craniomandibular Disorders. University San Pablo-CEU, Madrid, Spain; 2 Physical Therapist, Director and Professor. Master’s Degree Program in Orofacial Pain and Craniomandibular Disorders. University San Pablo-CEU, Madrid, Spain; 3 Physical Therapist, Department of Physical Therapy, Occupational Therapy, Rehabilitation and Physical Medicine, University Rey Juan Carlos Alcorcón, Madrid, Spain. Professor. Master’s Degree Program in Orofacial Pain and Craniomandibular Disorders. University San Pablo-CEU, Madrid, Spain; 4 MD, DMD, MS, Coordinator and Professor. Master’s Degree Program in Orofacial Pain and Craniomandibular Disorders. University San Pablo-CEU, Madrid, Spain

## Abstract

**Background:**

Myogenic temporomandibular disorders (MTMD) frequently coexist with other clinical conditions in the same individual. In the last decades, several authors have analyzed these comorbidities looking for the origin of this overlapping.

**Objetives:**

The aim of this study was to perform a comparative anaylisis between a group of patients with MTMD and a control group of dental patients without dysfunctional pathology to assess whether there are significant differences in the presence of systemic medical comorbidities between the two groups.

**Material and Methods:**

Restrospective epidemiological analysis, based on medical questionnaires in a group of 31 patients, women, aged from 24 to 58 (average 39.96 years), diagnosed with MTMD (Masticatory Myofascial Pain), with a control group with the same number of individuals, gender and age range to evaluate if there is a significant statistical difference in the presence of medical comorbidities in this group of patients with MTMD and if they are in a higher risk of suffering different pathological conditions.

**Results:**

It was found that the group affected by MTMD presented many more associated medical conditions than the control group: health changes during the last year, medical evaluations and treatments, presence of pain, sinus disease, tinnitus, headache, joint pain, ocular disorders, fatigue, dizziness, genitourinary disorders and xerostomia among others; and they were also in a higher risk to suffer other pathological entities as headaches and articular pain.

**Conclusions:**

These results reinforce our hypothesis that MTMD belong to a group of medical conditions triggered by a loss of equilibrium of the individual’s Psycho-Neuro-Endocrine-Immune (PNEI) Axis that produces alterations in the response against external stimuli in some genetically predisposed individuals. It is, therefore, necessary to change the way of diagnosing and managing these individual’s medical conditions, being mandatory to look from a more multidisciplinary perspective than the one we are currently offering.

**Key words:**Temporomandibular joint disorders, myofascial pain syndromes, comorbidity, chronic pain, health status, health questionnaire, self report.

## Introduction

Temporomandibular Disorders (TMDs) is a general term that includes a group of clinical conditions affecting the temporomandibular joint, the masticatory musculature and associated head and neck musculoskeletal structures. It affects 5-12% of the general population and is the second most frequent cause of musculoskeletal pain and limitation, only preceeded by low back pain. Myofascial Pain is the most prevalent TMD diagnosis and it is defined as “pain of muscle origin that is affected by jaw movement, function, or para-function, and replication of this pain occurs with provocation testing of the masticatory muscles spreading beyond the site of palpation but within the boundary of the muscle when using myofascial examination protocol” (1). Myofascial pain syndrome can be described as the sensory, motor and autonomic symptoms caused by myofascial trigger points. A myofascial trigger point is defined as a hyperirritable spot in a taut band of a skeletal muscle. The sensitive spot is painful on compression and can give rise to characteristic referred pain, referred tenderness, motor dysfunction and autonomic phenomena (2).

Myofascial TMD frequently coexists with other clinical entities (1,3). The OPPERA study concluded that people with a history of lower back pain at enrollment had a 50% greater incidence of TMD than people without history of low back pain; a history of genital pain symptoms was associated with 75% greater incidence of developing TMD; whereas irritable bowel syndrome predicted first-onset TMD after adjustment for demographic characteristics and pain disorders (3). Several names are used to describe this type of comorbidities: Medically Unexplained Symptoms, Unexplained Clinical Conditions (4), Functional Somatic Syndromes, Somatization Disorders, Polysymptomatic Somatizers, Somatization Spectrum Conditions, Psychosomatic Syndromes, Idiopathic Pain Disorders (5). Consequently, several authors have agreed to use the term Central Sensitivity Syndromes (6,7), including different systemic diseases such as fibromyalgia, TMD, bowel problems, urogenital problems, headaches or chronic pain in different body areas (7).

The aim of this paper is to analyze the relationship of Masticatory Myofascial Pain (MTMD) with other systemic conditions by means of an epidemiological retrospective cohort study.

## Material and Methods

Procedure: This epidemiological retrospective cohort study based on health questionnaires was conducted in 2015, about four years after the initial evaluation of the patients at the Dental Clinic of the University San Pablo-CEU in Madrid. The research project was approved by the Ethical Committee of the Master in OFP & TMD and all patients signed an Informed Consent Document. The study was conducted from the 66 variables ( Table 1) in the general health questionnaires of the medical history: general health, changes in general health in the last year, hospitalization in the last three years, present treatments, problems with prior dental treatments, present pain complaint, chest pain / angina, swollen ankles, fatigue, weight loss / night sweats, persistent or bloody cough, coagulation problems, sinusitis, difficulty swallowing, frequent nausea/vomiting, diarrhea / constipation / fecal blood, difficult urination / bloody urine, dizziness / vertigo, ringing in the ears, headaches / migraines, loss of consciousness, blurred vision, excessive thirst, frequent urination, dry mouth, jaundice, joint pain, stiffness, heart disease, heart attacks, cardiac birth defects, rheumatic fever, high blood pressure, tuberculosis / emphysema / other lung diseases, hepatitis / liver disease, stomach / ulcers, allergies, family problems of diabetes, heart disease or tumors, AIDS, tumors / cancer, arthritis / rheumatism, eye diseases, skin diseases, anemia, syphilis / gonorrhea, herpes, kidney disease, thyroid disease, diabetes, psychiatric treatment, radiotherapy, chemotherapy, artificial heart valves, joint prostheses, hospitalization, blood transfusions, surgery, pacemakers, contact lenses, drug abuse, medication use, smoking, alcohol consumption, current pregnancy, contraceptive use and other medical problems.

**Table 1:**
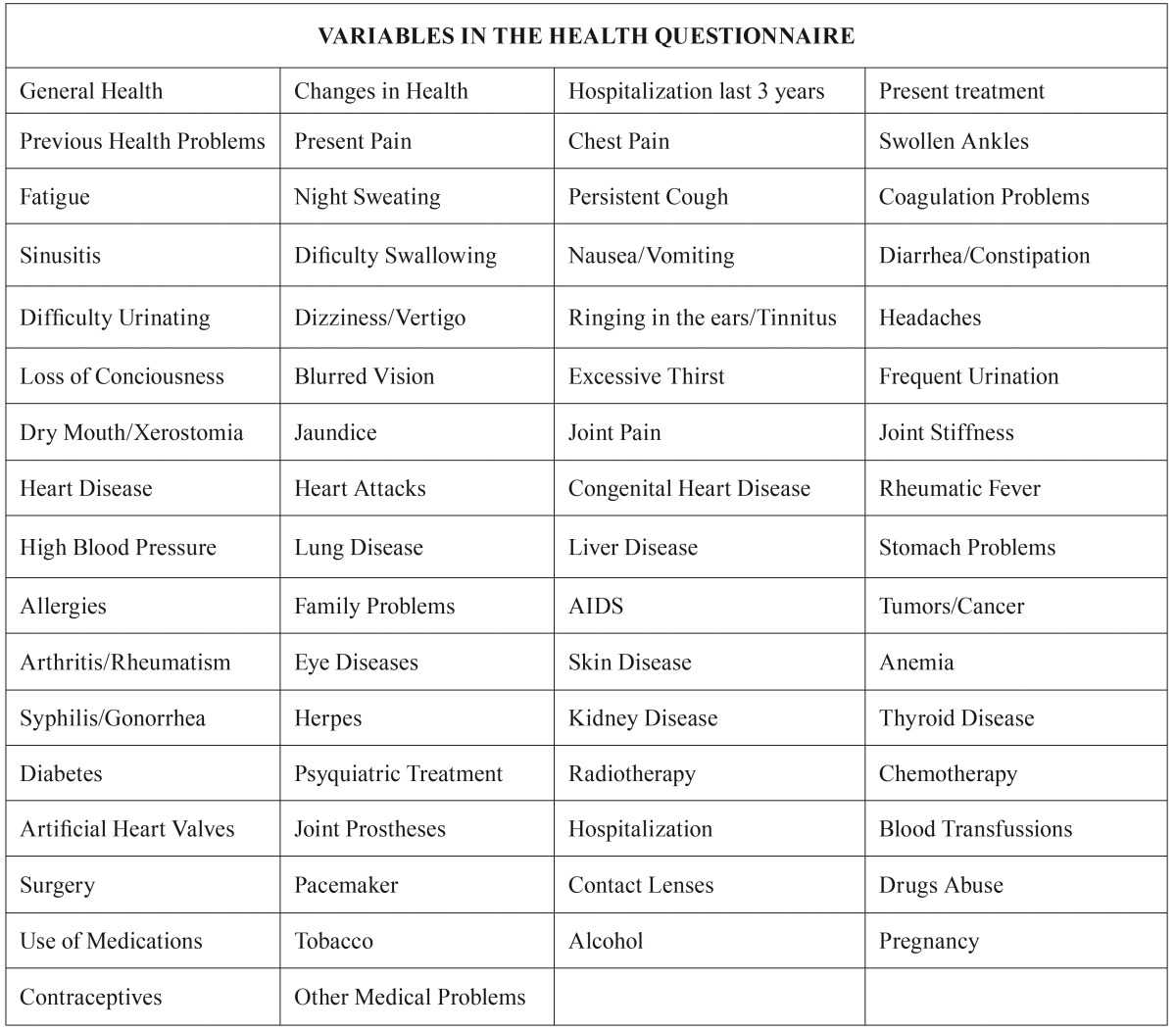
Procedures: variables in the health questionnaire.

**Figure 1:**
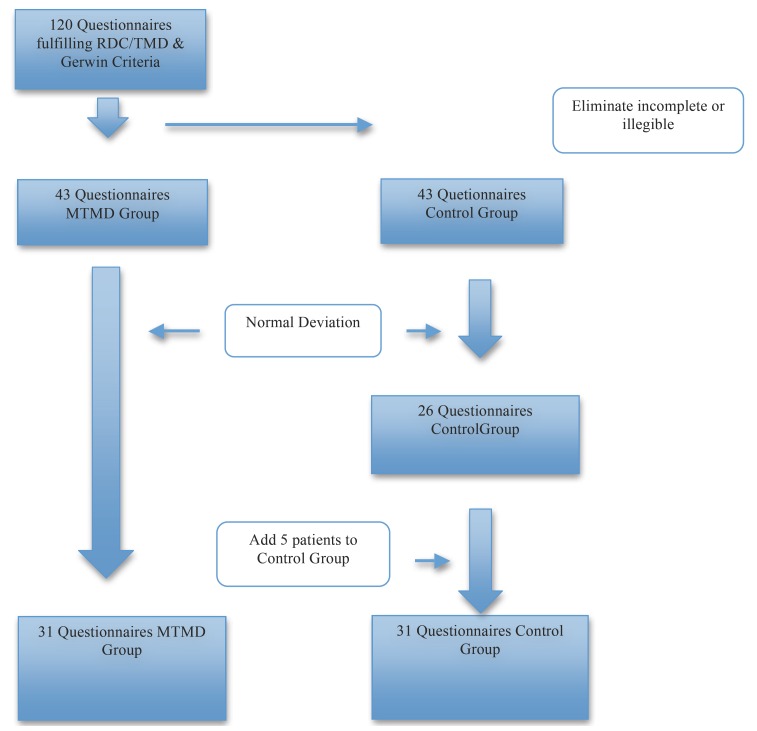
Sample.

Sample: The study group was composed of 120 Health Questionnaires of female patients who fulfilled the RDC/TMD criteria of myofascial pain (1). We excluded men from the study because the majority of patients affected by MTMD are women (3-7). Only complete and validated or confirmed by medical reports by corresponding complementary analytical test Health Questionnaires were included in this study, excluding those that were incomplete or contained any illegible answer and obtaining a from total of 43 patients who met all requirements. On the other hand, we selected another 43 Health Questionnaires of female patients who came to the university clinic for general dental practices (also meeting these criteria of validation and analytical confirmation) for the control group (Fig. 1).

Once the two initial groups (study and control) were selected, we performed the standard deviations of the total number of patients and of the two subgroups taking the age as variable. The average age in the MTMD group was 38.67 years, while in the control group it was 41.25 years, with a total average of 39.96 years for both groups. Within the standard deviation between 24 and 58 years old there were 31 patients in the TMD group and 26 in the control group. Patients that were not in this range were excluded from the study. We finally added 5 patients to the control group to equalize both groups in 31 subjects between 24 and 58 years.

Measures: All patients included in the MTMD group fulfilled the diagnostic criteria of the RDC/TMD (1) and also the Gerwin criteria for the diagnosis of myofascial trigger points (8) ( Table 2).

**Table 2:**
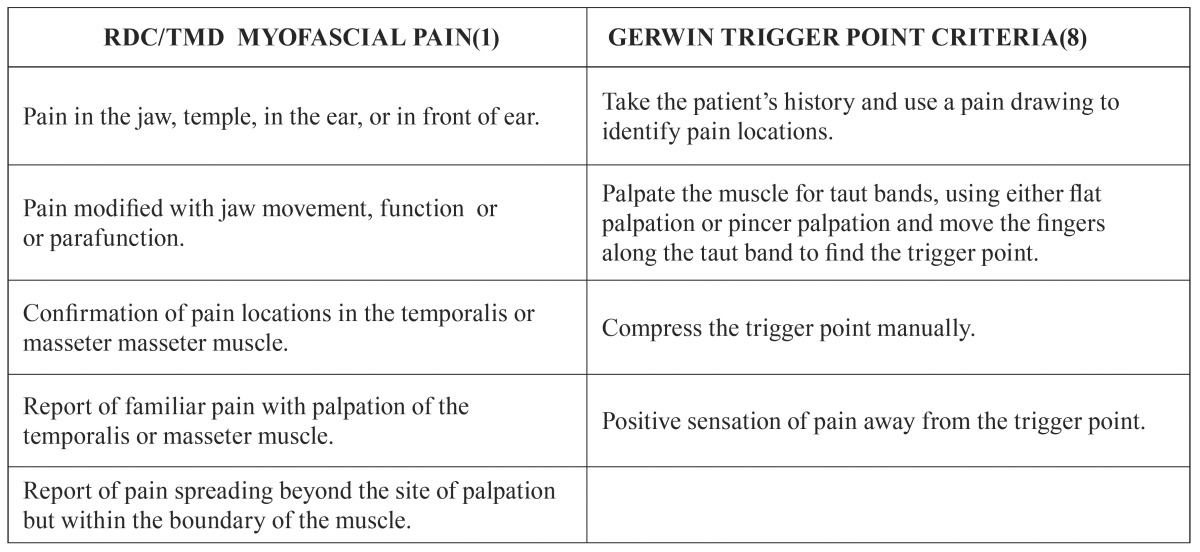
Measures: diagnostic criteria MTMD group.

Statistical analysis: Analysis of the relationship between all the qualitative variables and the group to which the individual belonged (MTMD or control group) was made to check whether there was a disproportion between the different categories of qualitative variables depending on the group of the individual using chi-square test of the SPSS program v. 2.0, performing contingency tables to cross each of the variables with the group variable (null hypothesis was proposed as independence between the variables and the group) to obtain the contrast p-value. It was considered that there were significant imbalances when p-values were less than 0.05; working, therefore with 95% confidence rate. Odd Ratio (OR) was also calculated to assess the risk estimation of the different variables in both groups.

## Results

**Figure 2:**
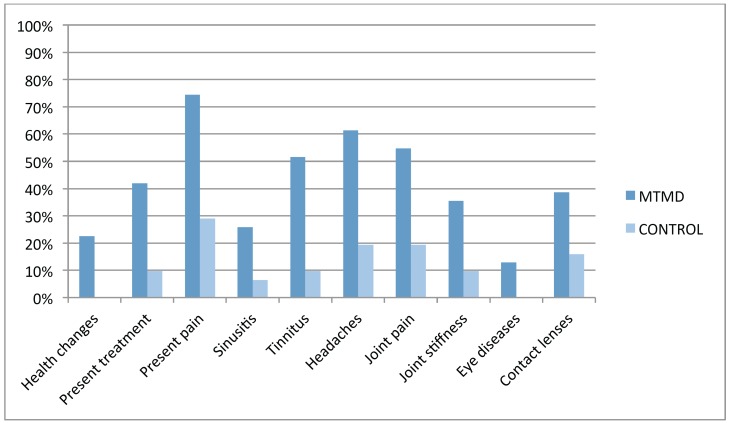
Distribution of medical conditions with statistical differences.

In the vast majority of variables, the patients in the study group (MTMD) suffered more medical comorbidities. Ten variables with statistical significance between both groups were found (Fig. 2): health changes during the last year (22.6% MTMD vs 0% control. *p*=0.005), present medical treatment (41.9% MTMD vs 9.7% control. *p*=0.004), present pain (77.4% MTMD vs 29% control *P*<0.001), sinusitis (25.8% MTMD vs 6.5% control. *p*=0.040), tinnitus (51.6% MTMD vs 9.7% control. *P*<0.001), headaches (61.3% DCM vs 19.4% control. *p*=0.001), joint pain (54.8% MTMD vs 19.4% control. *p*=0.004), joint stiffness (35.5% MTMD vs 9.7% control. *p*=0.016), eye disease (12.9% MTMD vs 0% control. *p*=0.050) and contact lenses (38.7% MTMD vs 16.1% control. *p*=0.043) ( Table 3)

**Table 3:**
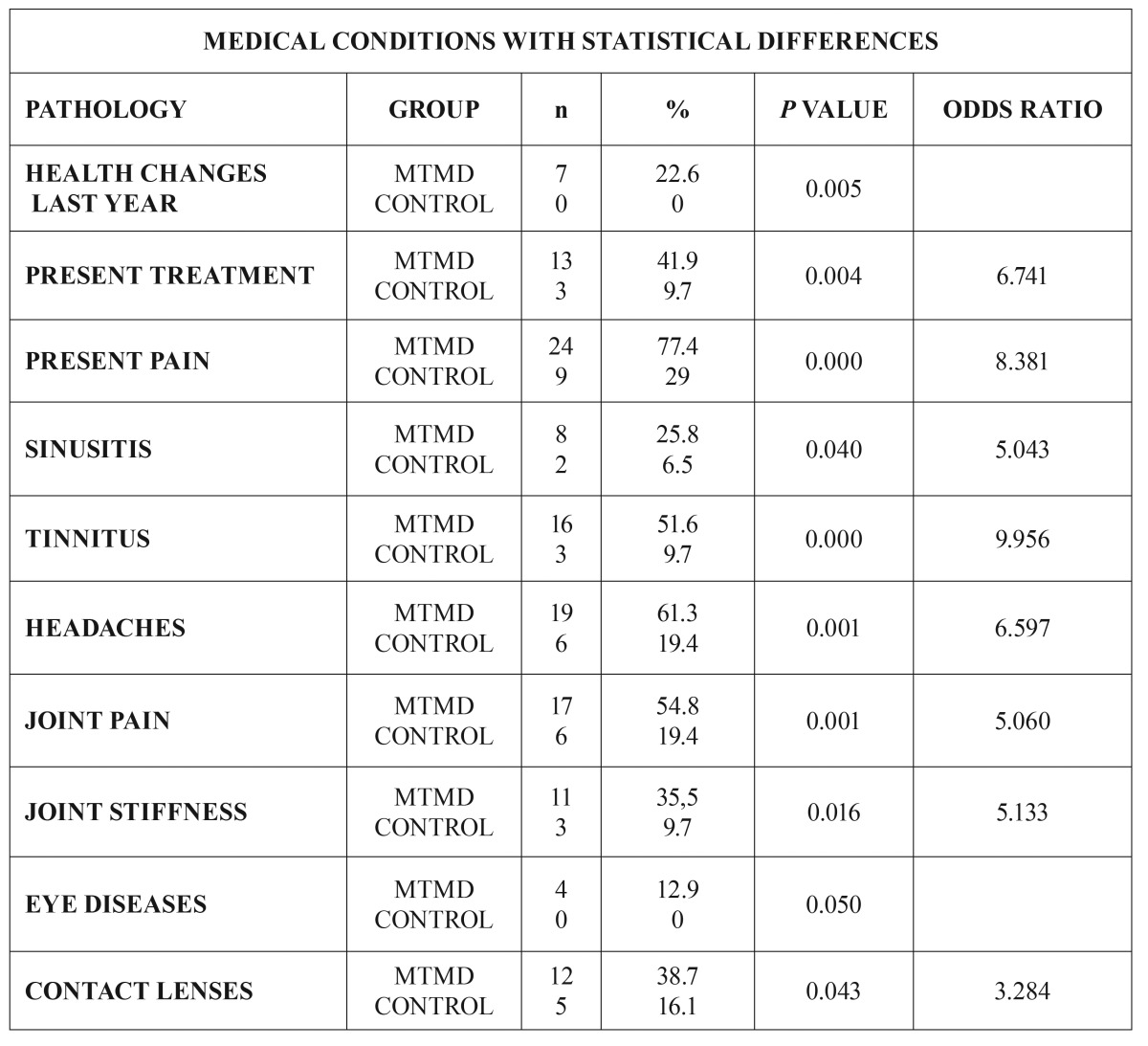
Medical conditions with statistical differences.

**Figure 3:**
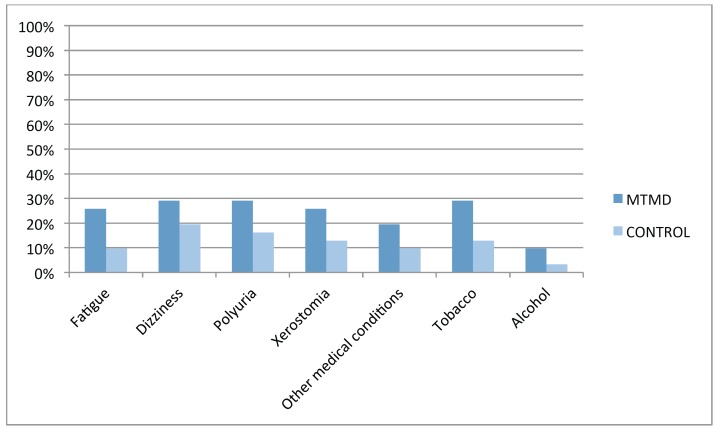
Distribution of other medical conditions.

Important, though not statistically significant, differences were observed in another 7 variables (Fig. 3): fatigue (25.8% MTMD vs 9.7% control. *p*=0.091), dizziness and vertigo (29% MTMD vs 19.4% control. *p*=0.277), poliuria/frequent urination (29% MTMD vs 16.1% control. *p*=0.181), xerostomia/dry mouth (25.8 % MTMD vs 12.9% control. *p*=0.168), other medical conditions (19.4% MTMD vs 9.7% control. *p*=0.236), smoking (29% MTMD vs 12.9% control. *p*=0.106) y alcohol consumption (9.7% MTMD vs 3.2% control. *p*=0.306) ( Table 4).

**Table 4:**
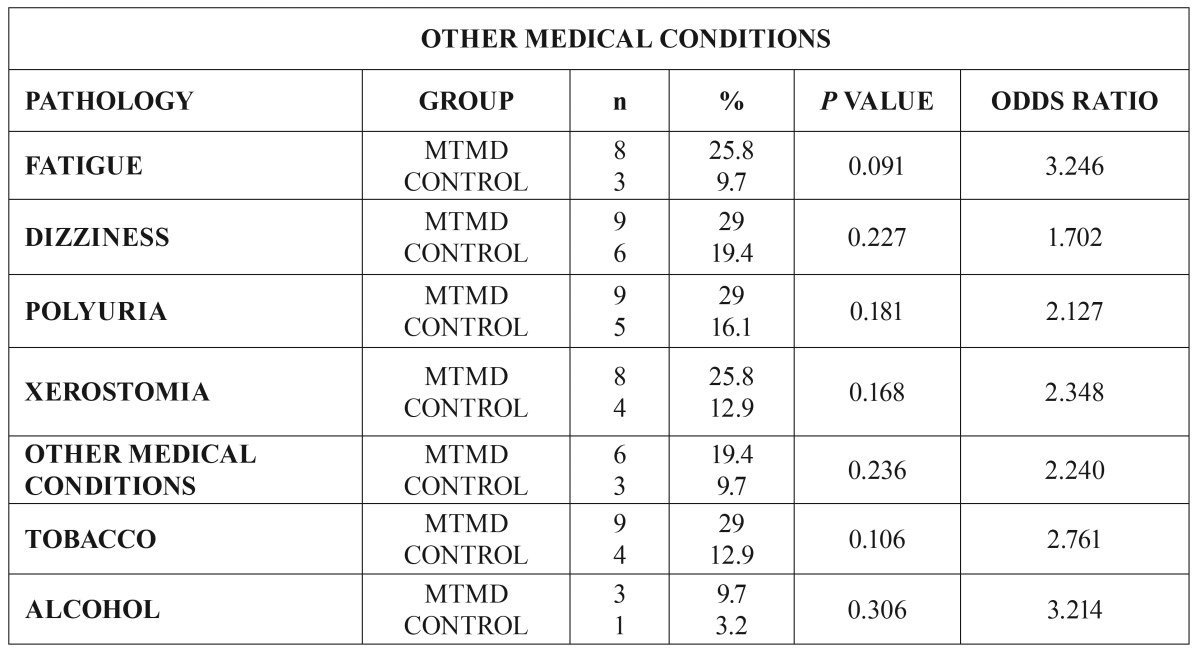
Other medical conditions.

Finally, after analyzing the OR, it was found that MTMD patents where in a higher risk of being under a higher number of medical treatments (OR 6.741), suffered more pain (OR 8.381), sinusitis (OR 5.043), tinnitus (OR 9.956), headaches (OR 6.597), joint pain (OR 5.060) and joint stiffness (OR 5.133).

## Discussion

Our results support the hypothesis that MTMD patients are at greater risk of suffering other medical conditions than the healthy population, which is in agreement with previous studies (9-12). We found 10 health variables showing statistical difference between the MTMD Group and the Control Group. The variable health changes during the last year includes patients diagnosed with a new medical condition or worsening of a prior existing condition confirmed by their physician and/or analytical report; this was present in 22.6% of patients in the MTMD Group versus none (0%) in the Control Group. Almost half (41.9%) of the MTMD patients were presently receiving another medical treatment versus 9.7% in the Control Group. This confirms the fact that MTMD is usually comorbid with other medical conditions (4,7,9-12) as fibromyalgia or chronic fatigue syndrome (6,8). 77,4% MTMD patients had present pain vs. 29% the Control Group; these data are very similar to those of Aaron *et al.* (9) and Hoffmann *et al.* (11). 25.8% of MTMD patients had a diagnosis of sinusitis (according to the Diagnostic Criteria of the ICHD-3β: 11.5.1: Headache attributed to Acute Rhinosinusitis (13), against a 6.5% of the Control Group; Charleston *et al.* (14) state the existence of possible pathophysiological interactions between MTMD and sinusitis, due to a hypersensitivity to external stimuli secondary to CNS sensitization (orofacial musculature and paranasal sinuses share a common innervation via V1, V2 and the sphenopalatine ganglion). More than half (51.6%) of the MTMD patients referred the presence of tinnitus compared with 9.7% in the Control Group; these figures are comparable with those of other similar studies (9-12). Tinnitus is described as “the subjective perception of sound in the absence of apparent acoustic stimuli” (15) and can be caused by otologic, neurological, traumatic, medicinal, nutritional, depressive and origin myogenic craniomandibular dysfunction pathologies according to Calderon *et al.* (15) These authors did not find any agreement among the different etiopathological hypothesis, but stating that tinnitus and chronic pain produce similar alterations in the Central and Peripheral Nervous System due to neuroplasticity and central sensitization. Teachey *et al.* (16), claim that there is a direct influence of MTMD in the presence of tinnitus due to the presence of myofascial trigger points in masseter and esternocleidomastoid muscles that improve with adequate physical therapy treatment of these trigger points. 61.3% of MTMD patients complain of Headache (19,4% in the Control Group). Headache diagnosis is made according to the ICHD-3β (13) for primary headaches (1,2,3,4 ICHD-3β) or those attributed to TMD (11.7 ICHD-3β). In 2013, Da silva *et al.* (17) stated that “TMD are an important comorbidity of migraine and may be clinically difficult to distinguish them from tension-type headache” (17). According to these authors, 50% of the population suffers from headache at some point in their life and TMD affects 40-60%, confirming the comorbidity. Articular Pathology: 54.8% MTMD vs 19.4% Control Group suffer Joint (Articular) Pain, and 35.% MTMD vs 9.7% Control Group feel Joint Stiffness. We included in this group those patients fulfilling the RDC/TMD Criteria of Artralgia and Myogenic TMD (1) and those with a written medical report regarding this pathology: Inoue *et al.* support the hypothesis that there is comorbidity between MTMD and TMJ Artralgia (18). Sipila *et al.* conclude that “masticatory muscle pain is associated with pain in the back, neck, shoulders, and other joints due to central sensitization” (19). Eye Disease: 12.9% of MTMD suffer from Eye Disease (with confirmed medical diagnosis) vs. 0% in the Control Group. 38.7% of MTMD patients use prescription glasses or contact lenses vs. 16.1% in the Control Group: Monaco *et al.* propose that this is due to an alteration of proprioception in the cervical muscles (20).

We have also found important, though not statistically significant, differences between both groups in some other variables: Fatigue, 25,8% vs. 9,7%. Chronic Fatigue Syndrome and Fibromyalgia (with medical confirmed diagnosis) were included in this group as they have been confirmed in many other studies (5,7,9-12,15,16,21). In 2012, Da Silva *et al.* presented a case control study to analyze the prevalence of orofacial pain in fibromyalgia patients. The conclusion was that the comorbidity between TMD and fibromyalgia is due to central sensitization, with high levels of substance *P* and growth factor causing a compromise in thalamic function (21). That same year, Fraga *et al.* concluded that MTMD should be included as diagnostic criteria in fibromyalgia (22). 29% of MTMD patients refer the presence of Dizziness/Vertigo vs 19.4% in the control group. Hubbard considers that these symptoms are due to cervical proprioceptive stimuli and they may be addressed with the therapeutic management of the head and neck trigger points (23). As it is shown in similar studies (9-12), genitourinary symptoms (chronic pelvic pain, interstitial cystitis, painful bladder syndrome and vulvodynia (24) are more prevalent (29%) in MTMD patients than in the control group. Rodríguez *et al.* in 2009 concluded that 13% of patients with TMD also suffer from cystitis and 20% of patients with vulvodynia present muscular pain (24). 25.8% MTMD patients refer xerostomia vs 12.9% in the control group. Xerostomia is defined as that “sense of lack of saliva, which may or may not coincide with the actual salivary flow volume” (25). According to Da Silva *et al.*, the volume of salivary flow in TMD patients is compromised due to central sensitization, hypothalamic function compromise or secondary to the pharmacologic treatment used in these patients (25). Smoking (more than 100 cigarettes a week): 29% of MTMD patients smoke in comparison with 12.9% in the control group (26). De Leeuw *et al.* included smoking as one of the main comorbidities of TMD (12) and so did the OPPERA Study (3). Burris *et al.* have recently confirmed that tobacco is statistically related to pain and psychological functioning of patients, causing increased fatigue and adversely affecting pain experience and quality of sleep (26).

It seems that the relation among all these pathologies goes well beyond that of functional neurological compromise or pain perception. Some other factors, as an excess of histamine (27) or the role of Na+ channels, especially Nav 1.9 in bowel function and orofacial pain have a significant influence. These channels are found in the soma of small and medium size peripheral neurons and in afferent fibers of the lip and dental pulp, showing the close relation between gastrointestinal syndromes and trigeminal pain (28). The relationship between Nav 1.7 and Nav 1.8 Na+ channels and chronic pain is presently a matter of research interest as well. Some mutations and phenotype variations are related with a higher expression of pain sensation (for example, the enzyme COMT contains at least five functional polymorphisms that significantly affect its activity (28,29). Scientific evidence is showing that all these entities (irritable bowel syndrome, interstitial cystitis, dyspepsia, fibromyalgia, TMD, chronic fatigue syndrome, among others) share common pathophysiological mechanisms leading to an altered pain perception (2,4-12,23,24,28-30) and cerebral activation models (29), peripheral immunity activation (4,27-30), post-infectious factors (29), neuroendocrine dysregulation (2,4-12,28-30) and genetic influence (28-30).

Other authors as Ablin and Clauw state that the main reason for the existence of these comorbidities and chronic pain syndromes is the disruption of the equilibrium of the PsychoNeuroEndocrineImmune Axis in genetically predisposed patients due to the influence of various stressing factors, proposing the concept of Central or Centralized Pain (30).

Our study has several limitations: the group size is smaller than in other similar studies (9-12), the questionnaire used for the study may not be the most specific for certain type of pathologies as functional digestive disorders and the population of the study is mainly (but not all) from downtown Madrid. It would be interesting to perform a multicentre study with a broader geographic distribution.

The analysis and discussion of our results with the appropriate literature allows us to formulate the Conclusion that MTMD belongs to a group of clinical entities that have in common the alteration of the functional equilibrium of the PsychoNeuroEndocrineImmune Axis in genetically predisposed individuals.

## Conclusions

The main conclusions of our study are:

1. Patients with MTMD present a higher number of medical comorbidities (present pain, sinusitis, tinnitus, joint pain, eye disease fatigue, vertigo/dizziness, functional genitourinary disorders, xerostomia) than the general population.

2. In these patients, these pathologies seem arise from the loss of functional equilibrium of the PNEI Axis due to a variety of stress factors. There seems to be a genetic predisposition to this sensitivity.

3. The relationship among all these pathologies seems to go beyond the mere presence of pain o the involvement of central sensitization but they should be included in the group of Central Sensitivity Syndromes, a collective term embracing a group of clinical problems that reflect the loss of equilibrium of the PsychoNeuroEndocrineImmune Axis in genetically predisposed sensitive individuals. Those individuals would present hypersensitivity in the reaction to a variety of internal or external stimuli that attempt to alter the homeostatic equilibrium of the human organism.

4. To correctly address these entities a paradigm shift is necessary to broaden the medical scope of diagnosis and treatment from the ancient mechanistic model to a modern interdisciplinary biopsychosocial approach.
